# Innovations in Biosensor Technologies for Healthcare Diagnostics and Therapeutic Drug Monitoring: Applications, Recent Progress, and Future Research Challenges

**DOI:** 10.3390/s24165143

**Published:** 2024-08-08

**Authors:** Mohamed Hemdan, Mohamed A. Ali, Ahmed S. Doghish, Sherif S. Abdel Mageed, Ibrahim M. Elazab, Magdy M. Khalil, Mostafa Mabrouk, Diganta B. Das, Alaa S. Amin

**Affiliations:** 1 School of Biotechnology, Badr University in Cairo (BUC), Badr City 11829, Egypt; mohamed.hemdan@buc.edu.eg (M.H.); mohamed.ahmed_ali@buc.edu.eg (M.A.A.); 2 Department of Biochemistry, Faculty of Pharmacy, Badr University in Cairo (BUC), Badr City 11829, Egypt; ahmed_doghish@azhar.edu.eg; 3 Biochemistry and Molecular Biology Department, Faculty of Pharmacy (Boys), Al-Azhar University, Nasr City 11231, Egypt; 4 Pharmacology and Toxicology Department, Faculty of Pharmacy, Badr University in Cairo (BUC), Badr City 11829, Egypt; sherif.abdelmeguid@buc.edu.eg; 5 Department of Biochemistry, Faculty of Pharmacy, Tanta University, Tanta 31527, Egypt; ibrahim.elazab@pharm.tanta.edu.eg; 6 Medical Biophysics, Department of Physics, Faculty of Science, Helwan University, Cairo 11795, Egypt; magdy.khalil@buc.edu.eg; 7 School of Applied Health Sciences, Badr University in Cairo (BUC), Badr City 11829, Egypt; 8 Refractories, Ceramics and Building Materials Department, National Research Centre, 33 El Bohouth St., Giza 12622, Egypt; mostafamabrouk.nrc@gmail.com; 9 Department of Chemical Engineering, Loughborough University, Loughborough LE11 3TU, UK; 10 Chemistry Department, Faculty of Science, Benha University, Benha 13511, Egypt; asamin2005@hotmail.com

**Keywords:** biosensors, healthcare applications, disease biomarker detection, therapeutic drug monitoring, nanotechnology integration

## Abstract

This comprehensive review delves into the forefront of biosensor technologies and their critical roles in disease biomarker detection and therapeutic drug monitoring. It provides an in-depth analysis of various biosensor types and applications, including enzymatic sensors, immunosensors, and DNA sensors, elucidating their mechanisms and specific healthcare applications. The review highlights recent innovations such as integrating nanotechnology, developing wearable devices, and trends in miniaturisation, showcasing their transformative potential in healthcare. In addition, it addresses significant sensitivity, specificity, reproducibility, and data security challenges, proposing strategic solutions to overcome these obstacles. It is envisaged that it will inform strategic decision-making, drive technological innovation, and enhance global healthcare outcomes by synthesising multidisciplinary insights.

## 1. Introduction

The healthcare landscape is evolving quickly, propelled by technological innovations, scientific advancements, and paradigmatic shifts, reshaping traditional models, methodologies, and modalities of patient care, disease management, and therapeutic interventions [1,2,3]. Currently, numerous digital biomedical tools are accessible for assessing different facets of human activity and capabilities. The global count of linked wearable gadgets is projected to surge from approximately 300 million in 2016 to more than 1 billion by 2024 [4]. Rapid technology advancement is fuelling the growth of digital hospitals, e-health, and telemedicine. The use of electronic health monitoring in patient diagnosis and treatment is growing dramatically as healthcare staff become scarcer. Wireless ubiquitous monitoring has become a key tool in the biomedical and healthcare industries in recent years [5,6,7].

A biosensor is an advanced analytical device designed with precision to detect subtle changes in complex biological processes, converting these delicate variations into clear electrical signals. Biosensors integrate molecular recognition elements (MREs) with transducers. MREs vary from cells, receptors, and antibodies to synthetic materials like Molecularly Imprinted Polymers (MIPs) and Peptide Nucleic Acids (PNAs). These MREs are categorised into affinity and catalytic groups; the former includes MIPs and antibodies, while the latter features cells and enzymes [8,9,10,11,12]. Enhancing biosensor durability involves using specialised enzymes and microbes. Notably, MIPs offer covalent and non-covalent variants, crucial for biomedical sensors that detect compounds such as herbicides [13,14], beta-estradiol sensors [15,16,17], and chloramphenicol sensors [18,19].

**Figure 1:**
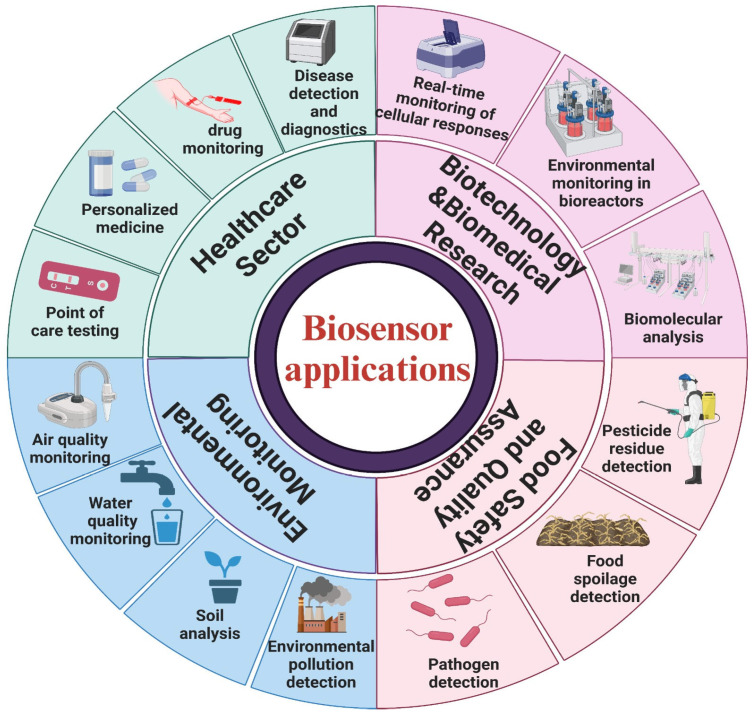
Various uses of biosensors in healthcare, biotechnology, environmental monitoring, and food safety.

With increasing significance and versatility, biosensors have found various applications, as graphically summarised in Figure 1. Traditional disease management and diagnostics approaches heavily relied on time-consuming clinical examinations, laboratory tests, imaging studies, and invasive procedures in healthcare. These methods often involved resource-intensive protocols and delayed interventions. However, introducing biosensors has transformed diagnostic approaches by enabling swift, precise, and cost-effective detection, identification, and monitoring of diseases in their early stages. This revolution has facilitated prompt interventions, personalised treatment plans, and enhanced patient outcomes [20,21,22,23,24].

In addition to diagnostics, biosensors are revolutionising therapeutic interventions, drug development, and personalised medicine initiatives by facilitating real-time monitoring tailored to individual patient profiles, genetic predispositions, and therapeutic responses. These biosensors enable precise pharmacokinetic/pharmacodynamic analyses, drug efficacy evaluations, and therapeutic monitoring protocols that empower clinicians to optimise drug dosages, minimise adverse effects, and enhance treatment efficacy while minimising therapeutic failures, drug resistance, and medication-related complications [25,26,27,28,29].

Emerging developments like wearable biosensors, point-of-care testing tools, implantable devices, telemedicine applications, and remote monitoring systems are broadening healthcare access, enhancing patient involvement, and reshaping healthcare delivery models to meet evolving societal demands, technological advancements, and healthcare priorities [30,31,32,33].

This review provides an insightful analysis of the progress in the contributions of biosensors in healthcare, emphasising their innovative applications and transformative potential. It also aims to inspire scientific inquiry, stimulate interdisciplinary collaboration, and inform evidence-based practices and policies that address complex healthcare challenges, promote global health equity, and enhance human well-being.

## 2. Fundamentals of Biosensors: Essential Components, Working Principle and Types

### 2.1. Essential Components of Biosensors

**Figure 2:**
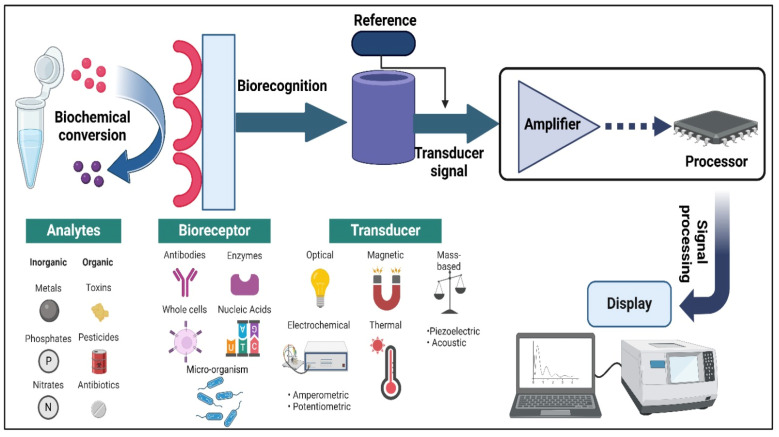
Key components of biosensors.

At the core of biosensors lies the bioreceptor, a pivotal element meticulously crafted to recognise and bind with the target analyte (Figure 2). This component fundamentally dictates the biosensor’s specificity and sensitivity, employing diverse arrays of bioreceptors, each endowed with distinct advantages [34]. Antibodies originating from the immune system exhibit exceptional specificity and can be tailored for various disease indicators, exemplifying their utility in biosensors [35]. Enzymes, acting as biological catalysts, undergo activity changes upon binding to the analyte, furnishing a measurable signal, particularly beneficial in biosensors designed for detecting specific enzymatic reactions [36]. Aptamers, short DNA or RNA sequences, showcase high target affinity and ease of modification, rendering them versatile in diverse biosensor applications [37]. Additionally, cells, whether as a whole or their components like organelles or membranes, offer a dynamic approach to biosensor design, responding to analytes by altering their electrical properties or signalling activity [38]. This intricate interplay of bioreceptors underscores the biosensor functionality’s nuanced and multifaceted nature.

In biosensors, the transducer is crucial as it transforms the biorecognition event into a measurable signal, which is essential for detecting the target substance. The choice of transducer depends on the bioreceptor type and the desired detection approach. Various transducers are commonly employed in biosensor design. Electrochemical transducers, encompassing amperometric, potentiometric, and conductometric devices, measure changes in electric currents, potential, or conductivity, with applications such as amperometric biosensors quantifying analytes through the measurement of current generated during electrochemical reactions [39,40,41]. Optical transducers gauge alterations in light intensity, wavelength, or polarisation upon interaction with the analyte, allowing biosensors to utilise fluorescence to detect specific molecules [42,43]. Thermal transducers measure heat changes associated with the biorecognition event, finding applications in biosensors focused on temperature-sensitive reactions [44]. Piezoelectric transducers sense minute changes in mass or pressure utilised in biosensors, capitalising on alterations in mass due to analyte binding [45]. Mass-sensitive transducers quantify changes in the mass of the bioreceptor induced by analyte binding, providing a sensitive detection method [46,47]. This array of transducers underscores the versatility and adaptability of biosensors across diverse detection modalities.

Electronics emerge as a critical component tasked with amplifying and processing signals generated by the transducer, often characterised by their initial weakness, necessitating enhancement for meaningful interpretation [48,49]. The electronics unit plays a pivotal role in refining the signal, filtering out noise, and converting it into a user-friendly format suitable for display or data analysis. Beyond this, biosensors may incorporate additional components to optimise their functionality for specific applications. Microfluidic channels regulating sample and buffer solution flows enhance precision and efficiency in analyte detection [50]. Integrated microchips reduce the size and improve the performance of biosensors, making them appropriate for portable diagnostics and point-of-care testing [51]. Incorporating biocompatible materials that do not interfere with biorecognition processes is crucial to maintaining the precision and dependability of biosensors in detecting target substances [52].

### 2.2. Working Principles of Biosensors

**Figure 3:**
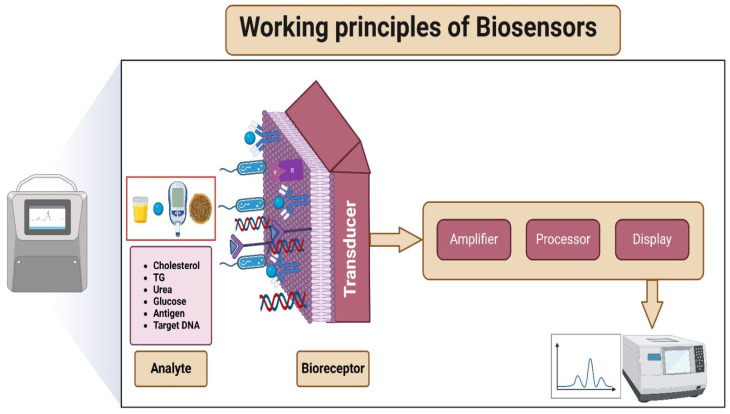
The operational principles and sensing mechanism employed by a biosensor.

Molecular recognition is the fundamental principle driving biosensor functionality, wherein the biorecognition element selectively engages with the target analyte (Figure 3). This specific and precise interaction is the cornerstone of the biosensor’s ability to identify and detect specific substances. A prime example of molecular recognition occurs in immunoassays, marked by the interaction between antigens and antibodies. Within this context, specialised proteins generated by the immune system selectively identify and attach to matching antigens, signalling the existence of the targeted analyte. This high degree of molecular specificity empowers biosensors with exceptional accuracy, enabling them to identify and capture particular molecules amid the complexity of a sample [53,54].

Following molecular recognition, the biosensors employ signal transduction mechanisms to transform the biological interaction into a detectable signal. This process is essential for converting the binding event into a format suitable for quantitative analysis. For instance, biosensors often employ enzymatic reactions to achieve this signal transmission. In these instances, when the analyte binds to the enzyme, it initiates a particular biochemical response that produces an electrochemical indication. This measurable signal provides a quantitative representation of the analyte’s presence, allowing for precise detection. Signal transduction mechanisms thus play a pivotal role in bridging the biological recognition event with a tangible output, facilitating the interpretation of biosensor results in a practical and meaningful way [55,56,57].

The analyte and sensor interactions are crucial in understanding how biosensors operate. When the analyte binds to the biorecognition component, it initiates a series of responses within the sensor [58]. For example, a glucose biosensor where glucose, the analyte, binds to an enzyme specifically designed for glucose recognition. This binding event sets off a responsive mechanism, manifesting as changes in electrical conductivity or the generation of a measurable signal. These responses are instrumental in the accurate detection and quantification of glucose levels [59,60].

A profound comprehension of these interactions between analyte and sensor forms the bedrock for the thoughtful design and optimisation of biosensors across diverse applications. Whether applied to monitor glucose levels in diabetic individuals or to identify specific biomarkers for disease diagnosis, the precision and sensitivity inherent in these interactions underscore the effectiveness and reliability of biosensor technology across a spectrum of fields.

### 2.3. Non-Specific Adsorption in Biosensors

Non-specific adsorption is the unwanted attachment of molecules to a surface and negatively impacts biosensor performance. It interferes with the desired interactions between molecules and surfaces, leading to inaccurate results and decreased sensitivity. There are two main approaches to reducing non-specific adsorption, namely, passive and active methods [61]. Passive methods involve modifying the surface properties to make it less attractive to non-specific molecules. This can be performed by coating the surface with a blocking agent, such as bovine serum albumin (BSA) or polyethylene glycol (PEG). Blocking agents create a steric barrier that prevents non-specific molecules from coming into close contact with the surface. Active methods involve using external forces to remove non-specific molecules from the surface. This can be performed by applying a shear force, such as with a liquid flow, or by using an electric field. The best approach for reducing non-specific adsorption will depend on the specific application. Passive methods are generally simpler to implement, but they may not be as effective as active methods. Active methods can be more complex, but they can be more effective in removing non-specific molecules [62,63].

### 2.4. Types of Biosensors

Biosensors play pivotal roles in contemporary diagnostics, highlighting various designs and functionalities. This thorough exploration of various biosensor categories sheds light on their underlying operational principles and presents various applications for each type. Table 1 delineates key applications for each biosensor type, underscoring their versatility and significance in healthcare. The following is an exploration of the diverse types of biosensors.

Enzymatic biosensors, anchored in enzymatic recognition elements, drive reactions that yield measurable signals, ensuring exceptional selectivity. In this process, enzymes facilitate reactions with the target analyte, producing substances that lead to measurable signals. Electrodes, commonly employed as transducers, then convert these signals into electrical responses [64]. A prime example of enzymatic biosensors is found in glucose monitoring, where glucose biosensors, prominently utilising glucose oxidase, are indispensable in managing diabetes. This enzyme catalyses the conversion of glucose, producing hydrogen peroxide, and the ensuing electrochemical signal, proportional to glucose concentration, facilitates real-time monitoring [65]. In a recent paper, Khalife et al. [66] developed an enzymatic biosensor for the quick and non-redox probe detection of vitamin K3. Engineered YaiB was fixed onto a carbon electrode, showing exceptional sensitivity and specificity. The biosensor facilitated rapid measurement in milk samples spiked with the compound, achieving a detection limit of 0.87 µM in buffer and 4.1 µM in milk [66].

Immunosensors utilise antibodies to detect proteins, pathogens, or specific molecules precisely. At their core, immunosensors operate through antibody-antigen interactions, where the existence of the targeted antigen induces binding, generating measurable signals detected through various transduction methods [67]. The swift, sensitive, and specific characteristics of immunosensors find exemplification in applications like pregnancy tests, where antibodies specific to human chorionic gonadotropin (hCG) facilitate its detection, demonstrating their efficacy in clinical diagnostics [68]. In recent research, as illustrated by Chaturvedi et al. [69], an advanced electrochemical immunosensor was developed using a nano-bio-inspired platform of reduced graphene oxide (rGO), polydopamine (pDA), and gold nanoparticles (AuNPs). These components were immobilised with anti-NS1 antibodies to detect the dengue NS1 protein. The sensor exhibits a linear response range from 1 ng mL^−1^ to 100 µg mL^−1^, with a sensitivity of 1.78 ng mL^−1^ mA^−1^, enabling early diagnosis of dengue virus infections.

DNA biosensors, employing nucleic acids as recognition elements, showcase remarkable precision in identifying specific DNA or RNA sequences. Their operational concept revolves around the pairing of target DNA with a matching probe, causing alterations in electrical or optical characteristics that convert into observable signals [70]. In the realm of genetic diagnostics, DNA biosensors play a pivotal role in detecting genetic mutations linked to diseases, with hybridisation events serving as sensitive indicators for early disease detection [71]. Recent research, as showcased by Ali et al. [72], introduces a label-free electrochemical DNA biosensor using a flexible indium tin oxide electrode to selectively detect *Shigella flexneri*. The study demonstrates a broad dynamic range, low detection limits, exceptional specificity, and successful deployment in spiked food samples.

Aptasensors, employing aptamers as the recognition elements, utilise single-stranded DNA or RNA molecules, offering remarkable versatility in target detection. Operating akin to immunosensors, aptasensors generate signals through the unique folding of aptamers upon binding to the target, which are then transduced for detection [73]. Their applications span a broad range, finding utility in both environmental monitoring and medical diagnostics due to their adaptability [74]. In recent research, Wu et al. [75] presented an extremely sensitive aptasensor for detecting deoxynivalenol. They utilised gold electrodes coated with polyethyleneimine-functionalised porous graphene oxide modified with gold nanowires. The sensor achieved a detection limit of 2.23 × 10^−9^ mg/mL through signal amplification using methylene blue-labelled zeolitic imidazolate framework-8.

Electrochemical biosensors, capitalising on changes in electrical properties, provide swift and sensitive measurements. These biosensors operate through biochemical reactions inducing measurable electrical changes, with electrodes facilitating detection through amperometric, potentiometric, or impedimetric methods [76]. Recent studies, such as those by Zhang et al. [77] showcase an electrochemical biosensor designed for highly sensitive detection of the HER2 protein. The biosensor utilises Apt-PNA probes and magnetic nanocomposites, achieving a detection limit of 4.1 femtograms per millilitre (fg·mL^−1^). The device showed encouraging outcomes in spiked serum samples.

Optical biosensors, engineered to detect variations in light properties, provide essential high sensitivity for real-time monitoring. Their working principle involves alterations in optical properties, such as absorbance or fluorescence, resulting from binding events at the recognition element and producing measurable signals [78,79]. Within this category, Surface Plasmon Resonance (SPR) biosensors play a prominent role and are widely utilised in drug discovery and environmental monitoring [80]. Recent research, exemplified by Yildizhan et al. [81] introduced a highly sensitive bioassay for detecting breast cancer-specific extracellular vesicles (EVs) in blood plasma. The study utilised a fibre-optic surface plasmon resonance (FO-SPR) biosensor, achieving limits of detection (LODs) of 2.1 × 10^7^ particles/mL for SK-BR-3 EVs and 1.1 × 10^8^ particles/mL for MCF7 EVs. This biosensor demonstrated outstanding sensitivity and specificity in clinical applications [81].

Piezoelectric biosensors, crafted to measure mass fluctuations, function by modifying the resonance frequency of a quartz crystal. The mechanism entails binding interactions at the recognition site that cause mass alterations on the crystal surface, resulting in frequency adjustments directly correlated to the analyte’s concentration. Notably, piezoelectric biosensors have proven invaluable for real-time, label-free monitoring of biomolecules, showcasing their exceptional sensitivity and versatility [82]. In their research conducted in 2023, Chen and Shi [83] developed a continuous flow immunoassay system that utilised a piezoelectric quartz crystal biosensor to detect alpha-fetoprotein (AFP) as a marker for tumours. The device demonstrated a quick response time of 12 min, detecting AFP at a rate of 45.9 nanograms per minute and detecting concentrations ranging from 18.8 to 1100 ng/mL. Although the specific details about the limits of detection and quantification were not specified, the biosensor underwent validation using clinical serum samples. The study confirmed that the biosensor did not react with lung cancer markers and strongly agreed with radioimmunoassay (RIA) findings. These findings indicate potential applications in cancer diagnosis and pharmaceutical research [83].

**Table 1:** Applications of diverse biosensor types in various healthcare scenarios.

Biosensor Type	Application	Biosensor Formulation	Ref.
Enzymatic Biosensor	Identification of the endocrine-disrupting chemical Bisphenol A (BPA).	Carbon paste electrode modified with Pd(II)-loaded PAMAM dendrimer and immobilised tyrosinase enzyme via crosslinking using glutaraldehyde.	[84]
	Glucose monitoring in diabetes patients.	A microneedle composed of polylactic acid (PLA), coated sequentially with gold nanoparticles (AuNPs), glucose oxidase (GOx), overoxidised polypyrrole (OPPy), and Nafion.	[85]
Immunosensor	Quick and precise detection of the antibiotic enrofloxacin (EF) in meat.	Anti-quinolone antibody on carbon electrodes with difloxacin-amino ferrocene electrochemical probe.	[86]
	Detecting SARS-CoV-2 in saliva using a combined vertical and lateral flow approach.	A container housing immunological reagents attached to magnetic beads alongside a lateral flow device featuring a polyester-based electrode, a magnet, and an absorbent pad.	[87]
DNA biosensor	Detecting Human papillomavirus-16 (HPV-16)	Glass electrodes coated with indium tin oxide (ITO), modified using graphene oxide and gold nanoparticles coated with silver, and immobilised with specific DNA probes for HPV-16 detection.	[88]
Colorimetric Aptasensor	Detection of Ochratoxin A (OTA).	DNA circuit driven by entropy (EDC), catalytic hairpin assembly (CHA), and magnesium ion-assisted DNAzyme catalysis (MNAzyme).	[89]
Electrochemical Biosensor	HER2 biomarker detection for breast cancer	Cd^2+^-aptamer@AMNFs@ZIF-67 nanocomposite	[90]
	Measuring levels of folic acid in expectant mothers	Dihydrofolate reductase (DHFR) anchored on a gold electrode modified with carbon-multiwalled carbon nanotubes (c-MWCNT) and titanium dioxide nanoparticles (TiO_2_NPs)	[91]
Optical biosensor	Detection of basal cell cancer	Arrays of one-dimensional photonic crystal (PC) structures linked with two metal–insulator–metal (MIM) plasmonic waveguides, utilising tapering techniques for enhanced alignment.	[92]
	Tracking biomarkers (MUC-1), utilising fluorescence imaging, and delivering targeted curcumin	A nanosystem of mesoporous silica, chitosan, and gold (MCM@CS@Au) targeted by aptamers for MUC-1 positive tumour cells.	[93]
Piezoelectric biosensor	Self-monitoring smart vascular grafts.	A biocompatible piezo composite with a low filler content of 5% volume and high piezoelectric sensitivity (g33~130 mV m N^−1^) features structured sodium niobate (NaNbO_3_) fibres embedded in an elastomeric matrix through dielectrophoresis.	[94]

## 3. Recent Advancements in Biosensor Technology

### 3.1. Nanotechnology in Biosensors

Incorporating nanotechnology into biosensors marks a revolutionary advancement, significantly impacting their design and performance. This paradigm shift introduces a diverse array of unique nanomaterials and structures that augment sensitivity and expand the horizons of detection limits. Nanoparticles, nanotubes, and nanowires have emerged as pivotal components, playing a transformative role in biosensing applications and contributing to remarkable progress in healthcare [95,96].

The integration of nanomaterials has ushered in a new era of biosensor development, significantly advancing healthcare technologies. Table 2 illustrates the transformative impact of nanotechnology on biosensor sensitivity and detection capabilities. Notably, nanoparticles such as gold nanoparticles have been crucial in enhancing the electrochemical performance of biosensors [97]. This enhancement is largely due to the high surface-to-volume ratios of nanoparticles, which facilitate extensive biomolecule immobilization and interactions. As a result, biosensors incorporating nanomaterials exhibit heightened sensitivity and improved efficiency in binding target molecules. The incorporation of these advanced materials has led to the creation of state-of-the-art biosensors that revolutionize healthcare by enabling precise detection of biomarkers associated with various diseases and providing accurate drug monitoring platforms [98].

**Table 2:** The utilisation of nano-enhanced biosensors across a spectrum of healthcare applications.

Biosensor Type	Used Nanoparticle	Application	Ref.
Label-free, ultrasensitive electrochemical biosensor	Gold nanoparticles (AuNPs)	Detection of transferrin (Tf), a crucial serum biomarker for atransferrinemia.	[99]
Electrochemical biosensor	Iron oxide nanoparticles capped with L-cysteine.	Determination of amino acids using L-Phenylalanine as the representative analyte.	[100]
Ultrafast and ultrasensitive DNA biosensor	Nanocubic architecture of MnFe@Pt crystals	Fast and precise determination of SARS-CoV-2 RNA via RNA reverse transcription to DNA (rtDNA).	[101]
Multiple-signal amplification PEC biosensor	Carboxylated graphitic carbon nitride (g-C_3_N_4_) paired with avidin-functionalised ruthenium-coated silica nanoparticles (Ru@SiO_2_).	Ultrasensitive detection of kanamycin residues in foods	[102]
Electrochemical biosensor	Multi-walled carbon nanotubes are functionalised with acid and tungsten wires coated with gold.	Rapid and easy detection of *Escherichia coli* (*E. coli*)	[103]
Optical biosensor	Gold nanoparticles (AuNPs) from green tea leaves	Biosensing of CD44 cancer biomarkers with improved sensitivity.	[104]
MicroRNA biosensor based on magnetic rod carbon paste electrodes	Carbon nanofibers combined with CuBTC-AIA (Copper-based Metal-Organic Framework) and Fe@rGO (Iron-reduced Graphene Oxide).	Detection of microRNA 155 for the diagnosis of breast cancer.	[105]
Nano immunosensor	Gold nanoparticles (AuNPs)	Detection and discrimination of Zika and Dengue viruses	[106]
Electrochemical aptasensor	Polypyrrole (PPy)-gold nanoparticles (AuNPs)	Detection of cardiac troponin I (cTnI) without labels for diagnosing acute myocardial infarction (AMI).	[107]
Fluorescence biosensor without enzymes or labels, operating on a ratiometric principle	DNA-silver nanoclusters (DNA-AgNCs)	Susceptible detection of tetracycline (TET) for antibiotic detection in food security	[108]
Dual-mode nano-biosensor combining ratiometric fluorescence and calorimetry	Carbon dots (BCDs) and Manganese dioxide nanosheets (MnO_2_ NSs)	Determination of *Staphylococcus aureus* *(S. aureus)*.	[109]
Surface plasmon resonance biosensor	TiO_2_/Au/graphene	A combined TiO_2_/Au/graphene layer-based surface plasmon resonance (SPR) sensor showed superior sensitivity (210 to 292.86 deg/RIU) for detecting cervical, skin, adrenal gland, breast, and blood cancer cells.	[110]

### 3.2. Integration with Wearable Devices

Integrating biosensors with wearable devices is revolutionising healthcare, bringing about continuous monitoring and personalised insights. Seamlessly woven into everyday attire, wearable biosensors facilitate real-time data acquisition, empowering proactive health management [111]. In a groundbreaking development, Li et al. [112] unveiled a novel wearable device for ongoing surveillance of the highly transmissible severe acute respiratory syndrome coronavirus 2 (SARS-CoV-2) and its mutations. This innovative method utilises 2D MXene-graphene composite films in a flexible field-effect transistor (FET) sensor. Embedded on masks and enhanced with Bluetooth connectivity for wireless data transfer, this sensor exhibits remarkable responsiveness to influenza and SARS-CoV-2 viruses in airborne environments and exhaled breath. This innovative wearable biosensor addresses the pressing need for continuous monitoring tools amid the ongoing challenges posed by respiratory viruses, particularly SARS-CoV-2.

In a separate study, Li et al. [113] pioneered a point-of-care (POC) sensing platform for diagnosing early chronic kidney disease (CKD). Employing reduced Graphene Oxide/Polydopamine-Molecularly Imprinted Polymer (rGO/PDA-MIP) with surface-molecular imprinting, the platform detects CKD biomarkers (creatinine, urea and human serum albumin) simultaneously. It integrates a multi-channel electrochemical POC readout system utilising differential pulse voltammetry, achieving an unmatched femtomolar-level limit-of-detection for all biomarkers. Clinical validation in serum and urine demonstrates efficacy, emphasising its cost-effectiveness, user-friendliness, and rapid sample-to-result time. The platform holds promise for resource-limited settings in CKD diagnosis and progression tracking.

Additionally, Yuksel et al. [68] developed an automated electrochemical point-of-care biosensor for detecting human chorionic gonadotropin (hCG) in urine, a critical biomarker for early pregnancy. The biosensor utilised custom gold and Ag/AgCl electrodes, optimised using COMSOL Multiphysics^®^ 5.2a software. DSP self-assembled monolayers were employed for covalently immobilising anti-hCG-beta antibodies, facilitating a sandwich assay with anti-hCG alpha-HRP. The device featured an innovative agitation design to accelerate surface reactions, enabling rapid detection of very low hCG concentrations. This research highlights the biosensor’s potential as a precise and efficient tool for early pregnancy diagnosis in clinical settings.

### 3.3. Miniaturisation and Portability

The evolution of biosensor technology has witnessed a transformative trend driven by advancements in microfabrication techniques, propelling the miniaturisation of biosensors to the forefront. This paradigm shift, characterised by reduced dimensions and enhanced portability, has profound implications for diagnostics, particularly in point-of-care applications. In this comprehensive exploration, we delve into the intricacies of miniaturisation and portability in biosensors, unveiling their potential through recent studies and innovations [114].

The miniaturisation of biosensors is closely linked to advancements in microfabrication techniques, enabling precise engineering of miniature structures and components. Microelectromechanical systems (MEMS) and microfluidics are pivotal in shaping biosensors at micro and nanoscales. MEMS principles involve the incorporation of mechanical elements, actuators, sensors, and electronics on a microscopic scale, allowing for susceptible and compact biosensor designs. Microfluidics principles focus on manipulating small fluid volumes within microchannels, facilitating controlled sample delivery and reaction kinetics in biosensing applications [115].

Miniaturised biosensors excel in providing rapid analyses with significantly reduced sample volumes, leveraging their inherent advantages of accelerated reaction kinetics for quicker response times. This attribute is vital in point-of-care settings, prioritising timely results. Working with small sample volumes offers significant benefits, particularly when samples are scarce. The compact size of miniaturised biosensors makes them well-suited for point-of-care use, allowing healthcare providers to conduct immediate analyses and make timely decisions and interventions [116].

## 4. Biosensors Application in Disease Detection

Biosensors are crucial in transforming disease detection and have become essential instruments in healthcare, as seen in Table 3. This section explores the diverse applications of biosensors in diagnosing various diseases, highlighting their versatility and significant impact on early detection and effective disease management. In the current healthcare landscape, where precision and timeliness are crucial, biosensors have emerged as invaluable contributors, providing revolutionary insights that transform diagnostics and enable proactive and personalised patient care. With their innovative capabilities, biosensors serve as essential allies, facilitating the early detection of diseases and enabling timely and targeted interventions. Their influence spans diverse medical domains, showcasing the versatility of biosensors as crucial assets in advancing healthcare outcomes.

### 4.1. Biosensors in Cancer Detection

Biosensors, utilising molecular recognition principles with antibodies or aptamers, are crucial for cancer diagnosis. They employ diverse sensing mechanisms such as optical (e.g., SPR), electrochemical, and mass-based (e.g., QCM) biosensors, enabling accurate measurement of cancer indicators such as prostate-specific antigen (PSA) or facilitating continuous monitoring of breast cancer biomarkers. These sensors are pivotal in converting specific interactions into detectable signals in diverse contexts, providing essential live data for efficient cancer diagnosis and therapy [117,118].

In a recent research paper, Wan et al. [119] explored innovative methods for detecting advanced breast cancer using biomarkers found in saliva. They developed specialised one-time-use strips that achieved highly sensitive detection levels, detecting HER2 and CA15-3 biomarkers at concentrations as low as 1 femtogram per millilitre. By employing a synchronised double-pulse technique, the study showcased swift testing speeds (under 15 milliseconds) and minimal sample needs (3 microliters of saliva). These advancements suggest a potential revolution in public health practices due to their easy-to-use design.

### 4.2. Biosensors in Infectious Disease Detection

Biosensors play a crucial role in diagnosing infectious diseases by using specialised recognition components such as antibodies or nucleic acids to interact with molecules associated with pathogens selectively. Immunosensors rely on antibodies binding to pathogen antigens, triggering measurable signals, while nucleic acid sensors detect genetic material through methods like PCR. Whole-cell-based mechanisms employ engineered living cells for signal production. Biosensors, adaptable to emerging threats, were notably used in colourimetric biosensors for visual detection of SARS-CoV-2 RNA during the COVID-19 pandemic [120,121].

**Figure 4:**
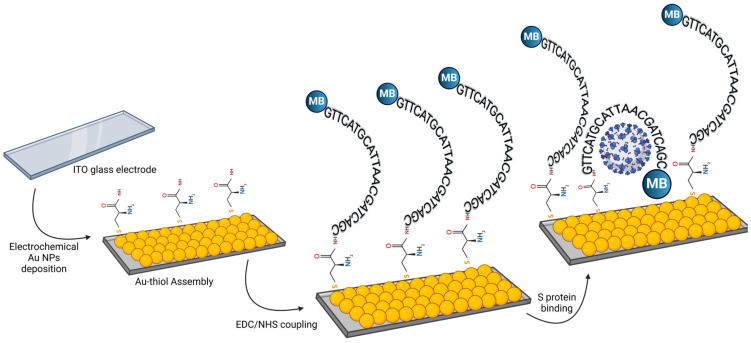
Schematic representation of the aptamer-based biosensor formulation and detection mechanism for COVID-19.

In a groundbreaking 2024 study, Khan et al. [122] developed an aptamer-based biosensor for rapid virus detection, significantly advancing large-scale COVID-19 identification. The biosensor formulation used a 60-base DNA aptamer targeting the S protein, immobilised on a gold nanoparticle-modified electrode with methylene blue (MB) as the redox indicator (Figure 4). Computational approaches identified the GLN104-ASP115 pocket as the aptamer’s binding site. The biosensor exhibited a broad linear detection range (10 pM to 6 nM) and a 91.1 pM limit of detection using differential pulse voltammetry. Testing in human saliva and serum demonstrated its capability for direct, single-step detection of viral proteins, highlighting its potential for COVID-19 and other viral agents.

### 4.3. Biosensors in Diabetes Management

Biosensors have revolutionised diabetes management by providing real-time monitoring, reducing reliance on invasive methods. Continuous glucose monitoring (CGM) systems lead the way in this advancement, seamlessly incorporating biosensor technologies to provide a constant flow of glucose information. This ongoing monitoring enables people to make informed choices regarding insulin doses and lifestyle changes instantly [123].

In an innovative 2024 research led by Li et al. [124], they developed a wearable microneedle sensor designed for continuous glucose monitoring (CGM). The device includes a microneedle electrochemical biosensor with three electrodes and a fully integrated radio-chemical analysis system. By electrodepositing Prussian blue (PB), crosslinking glucose oxidase (GOx), and utilising chitosan to form a 3D network with glutaraldehyde (GA), the sensor demonstrated long-term functionality in diabetic rats. The PB-based CGM system exhibited excellent sensitivity, stability, and resistance to interference, accurately monitoring glucose levels in the interstitial fluid (ISF) in real-time during experiments on diabetic rats. This study underscores the feasibility and promising clinical application of PB-based CGM for managing diabetes effectively. While biosensor advancements in diabetes management are promising, challenges such as accuracy, user adherence, and cost-effectiveness persist. Future directions involve refining sensor accuracy, improving data interpretation algorithms, and addressing issues of affordability and accessibility.

### 4.4. Biosensors in Neurological Disorder Detection

Biosensors have become transformative tools in early neurological disorder diagnosis, particularly in Alzheimer’s and Parkinson’s diseases. These devices, leveraging molecular recognition capabilities, offer unparalleled precision in detecting disease-associated biomarkers, surpassing traditional diagnostic methods. Besides Alzheimer’s, biosensors are crucial in detecting Parkinson’s disease early, facilitating timely identification and personalised treatment initiation. The accuracy and adaptability of biosensors offer a valuable understanding of the intricate realm of neurological conditions, highlighting the importance of early detection and specific interventions [125,126].

In a 2023 study by Altay et al. [127], a low-cost disposable immunosensor was created for the quick and sensitive measurement of the Aβ42 protein linked to Alzheimer’s disease. Though Electrochemical Impedance Spectroscopy (EIS) and Cyclic Voltammetry (CV), the immunosensor exhibited a linear detection span from 1 to 100 picograms per mL with a minimal detectable level of 0.37 picograms per mL, suggesting feasible clinical use for assessing Aβ42 in human blood serum. In a recent 2024 investigation led by Karaboğa et al. [128], an innovative biosensing strategy employed a quartz tuning fork (QTF) for the first time to develop an immunosensor targeting alpha-synuclein protein (SYN alpha), a potential biomarker for Parkinson’s disease. The biosensor, utilising a self-assembled monolayer and gold nanoparticles on QTFs, exhibited a selective detection range from 1 to 500 nanograms per mL and a low detection limit of 0.098 nanograms per mL. Significantly, the QTF-based biosensor effectively captured SYN alpha in cerebrospinal fluid samples, achieving recovery rates between 92% and 104%.

### 4.5. Biosensors in Cardiovascular Disease Detection

Cardiovascular diseases (CVDs) remain a major global health concern, necessitating sophisticated diagnostic instruments for timely intervention. Biosensors are crucial in quickly identifying cardiovascular conditions, especially myocardial infarction (MI) and heart failure. Representing a shift in cardiovascular disease diagnosis, biosensors offer swift and precise identification of specific biomarkers associated with cardiac events. Their impact is essential for timely interventions, leading to substantial improvements in patient outcomes and relieving the burdens on healthcare systems. The timely and accurate diagnosis of myocardial infarction, a critical aspect of the intervention, is facilitated by biosensors known for their sensitivity and specificity in detecting cardiac biomarkers related to MI [129,130].

In a study by Li et al. [131], they introduced an integrated microfluidic platform (IMS) designed for swift measurement of biomarkers associated with cardiovascular diseases (NT-proBNP, fibrinogen, cTnI, and CRP). Using aptamer-coated interdigitated electrodes and integrated circuits, the system enabled effective plasma filtration, quantification, and detection in just 15 min. This technology shows promise for assessing CVD risk and personalised medicine, achieving reasonable accuracy (>80%) in artificially spiked blood samples. In another study by Hui et al. [132], myocardial infarction (MI) diagnosis is addressed. Existing techniques lack early-stage sensitivity, prompting a focus on blood biomarkers. The study develops an interdigitated electrode sensor for troponin I (cTnI) detection, using aptamer-gold-cTnI-antibody sandwich patterns, achieving a 100 aM limit of detection and superior nonfouling in serum, offering potential for novel MI assays.

### 4.6. Biosensors in Autoimmune Disorder Detection

Biosensors are essential in diagnosing autoimmune disorders, specifically in conditions such as systemic lupus erythematosus (SLE) and rheumatoid arthritis (RA). Biosensors detect autoantibodies with high sensitivity and specificity, providing a targeted approach for early diagnosis. In autoimmune disorders, biosensors likely employ strategic recognition elements to capture disease-specific autoantibodies, utilising nanomaterials or surface modifications for enhanced sensitivity. The binding of autoantibodies initiates a measurable signal, allowing for quantification through analytical techniques. The ability to detect autoantibodies at low concentrations highlights biosensors’ potential for accurate and early diagnosis [133,134].

Focusing on RA and SLE, biosensors offer a transformative approach to identifying distinct autoantibodies associated with these conditions. Early detection through biosensors enables timely interventions, enhancing symptom control and preventing irreversible tissue damage [135]. In a study by Chen et al. [136], a novel method for rheumatoid arthritis (RA) detection was introduced using bacteriorhodopsin as a photoelectric transducer. The biosensor, employing a PM monolayer-coated electrode and a citrullinated-inter-alpha-trypsin inhibitor heavy chain 3 (ITIH3) 542–556 peptide, showed significantly reduced photocurrents with RA-patient serum compared to healthy controls. Labelling with anti-IgA-AuNP further amplified the distinction, demonstrating a close correlation with commercial assays, offering a sensitive and specific RA diagnosis in a single- or two-step mode. In another study by Jupe et al. [137], a decentralised digital approach utilising a biosensor for systemic lupus erythematosus (SLE) patients was implemented. Machine learning evaluated patient-reported outcomes (PROs), quality of life (QOL), and biometric data to forecast potential disease exacerbations. Digital surveys and smartwatches, acting as biosensors for biometric monitoring, collected data from 550 participants. The study resulted in a 26-feature model accurately distinguishing disease flare risks, facilitating proactive screening for clinical assessment.

### 4.7. Biosensors in Respiratory Disease Detection

Biosensors are pivotal in the field of respiratory illnesses, providing a revolutionary method for detection and treatment. The mechanism of biosensing involves the precise identification of biomarkers, often present in exhaled breath, through tailored recognition elements. In chronic obstructive pulmonary disease (COPD), biosensors excel in differentiating specific biomarkers associated with the condition, enabling accurate disease identification and personalised treatment strategies. This capability is especially vital in pulmonary fibrosis, where biosensors aid in the early identification of targeted biomarkers, paving the way for timely interventions and enhanced patient outcomes. The non-invasive nature of biosensor-driven diagnostics, combined with their capacity to interpret complex biomarker profiles, establishes them as essential instruments in advancing respiratory healthcare [138,139].

In a groundbreaking study by Qian et al. [140], a microdroplet analysis platform was developed as a biosensor, combining rolling circle amplification (RCA) and surface-enhanced Raman spectroscopy (SERS). This innovative, technology-enabled, accurate detection of miRNA-21 and miRNA-155 in the serum of patients with idiopathic pulmonary fibrosis (IPF) demonstrates high sensitivity and specificity with very low detection limits. The integrated microfluidic system addressed challenges related to SERS reproducibility. This biosensor, when used alongside chest computed tomography, emerged as a supplementary diagnostic tool for assessing IPF risk and monitoring miRNA levels in real-time during chemotherapy, highlighting its potential for versatile miRNA detection across different diseases [140].

**Table 3:** Examples of key biosensor applications in diverse disease diagnosis.

Target Disease	Biosensor	Transducer Type	Application	LOD	Linear Range	Ref.
Prostate cancer	Maackia amurensis (MAA) lectin immobilised on gold-interdigitated microelectrodes	Electrochemical	Accurate identification of cancer-linked abnormal glycosylation in prostate-specific antigen (PSA).	3.574 pg/mL	0.01–100 ng/mL	[141]
	Gold nanospikes (AuNS) arranged on a quartz crystal microbalance (QCM).	Electrochemical	Detection and assessment of prostate-specific antigen (PSA) for prostate cancer screening	24 pg/mL	0.1–100 ng/mL	[142]
Colon and rectal cancer	Electrochemiluminescence immunosensor with Ru@TiO_2_-MXene as the energy donor and Pd@UiO-66-NH_2_ as the energy acceptor	ECL-RET (Electrochemiluminescence Resonance Energy Transfer)	Detection of carcinoembryonic antigen (CEA) for diagnosis of colon and rectal cancer	2.65 fg/mL	1 × 10^−5^–80 ng/mL	[143]
Colorectal cancer	An electrochemical immunosensor employing nucleic acid aptamer recognition and silver deposition	Electrochemical	Identification of epidermal growth factor receptor (EGFR) for colorectal cancer diagnosis	0.06 ng/mL	1 to 1000 ng/mL	[144]
COVID-19 and Influenza	Liquid-gated graphene field-effect transistors (GFETs) with quadruple architecture and individual functionalisation	Electrical	Rapid and ultraprecise differentiation and sensing of influenza and SARS-CoV-2 surface proteins	~50 ag/mL, or 88 zM for COVID-19 and 227 zM for Flu	N/D	[145]
Diabetes	Non-invasive tear glucose biosensor based on a photoelectrochemical probe with optical fiber	Photoelectroch-emical	Non-invasive glucose detection.	4.1 nM	10 nM to 100 μM	[146]
	Polyaniline/nickel oxide nanohybrid modified graphite sheet (PANI/NiO/GS) biosensor	Electrochemical	Non-enzymatic electrochemical detection of methylglyoxal in human saliva.	2.64 nM	1 to 10 μM	[147]
	Phenylboronic acid (PBA)-based glucose biosensor with HCNT/PEDOT:PSS dual conductive structure	Electrochemical	Glucose detection with exceptional sensitivity, stability, and wide linearity range	N/D	0.20 mM to 2.0 mM	[148]
Alzheimer’s disease	Label-free impedimetric immunosensor using indium tin oxide polyethylene terephthalate (ITO-PET) electrodes modified with anti-Aβ42 antibodies	Electrochemical	Fast, specific, and highly sensitive quantitative assessment of Aβ42 protein for diagnosing Alzheimer’s disease	0.37 pg/mL	1–100 pg/mL	[127]
	A dual “turn-on” fluorescence biosensor using aggregation-induced emission fluorogen (AIEgen)-labelled oligonucleotide (TPET-DNA) probes attached to cationic dextran-modified molybdenum disulfide (TPET-DNA@Dex-MoS2).	Fluorescence	Fast, specific, and highly sensitive quantitative analysis of miR-125b for diagnosing Alzheimer’s disease and monitoring in real-time in PC12 cells and brain tissues of a mouse model of Alzheimer’s disease.	N/D	N/D	[149]
Parkinson’s disease	A biosensor based on a thin-film transistor (TFT) utilising indium gallium zinc oxide (IGZO) thin film technology	Electrochemical	Early detection of Parkinson’s disease using surface-functionalised IGZO TFT biosensor	N/D	1 pg mL^−1^ to 100 ng mL^−1^	[150]
Cardiovascular disease	An electrochemical immunosensor utilising nanostructured molybdenum tetraselenide reduced graphene oxide (nMo_3_Se_4_-rGO) for detecting cardiac troponin I (cTnI), offering a broader linear range, increased sensitivity, and lower detection limit.	Electrochemical	Early detection of cardiovascular disease using nMoSe-rGO-based biosensor for cTnI detection	1 fg mL^−1^	1 fg mL^−1^–100 ng mL^−1^	[151]
Rheumatoid arthritis (RA)	Tilted-fiber Bragg grating (TFBG) biosensor bonded with cyclic citrullinated peptide (CCP) antigens and antibodies	Optical	Laboratory-based detection of rheumatoid arthritis (RA) using cyclic citrullinated peptide (CCP) antibodies and antigens	1 ng/mL	N/D	[152]
	Magnetic microbeads functionalised with Neutravidin (NA-MBs) and modified with biotinylated anti-double-stranded DNA (dsDNA) for amperometric detection of anti-dsDNA autoantibodies (IgG, IgA, and IgM AAbs).	Amperometric	Detection of anti-dsDNA autoantibodies in the sera of rheumatoid arthritis patients	0.3 IU mL^−1^	1–200 IU mL^−1^	[153]
Chronic obstructive pulmonary disease (COPD)	Point-of-care biosensors for the multiplexed detection of interleukin (IL)-6, IL-8, matrix metalloproteinase (MMP)-8, MMP-9, C-reactive protein (CRP), tumour necrosis factor-alpha (TNF-α), and neutrophil elastase (NE)	Electrochemical	Identification of protein biomarkers in saliva and sputum for the management of chronic obstructive pulmonary disease (COPD).	N/D	N/D	[139]

## 5. Biosensors in Therapeutic Drug Monitoring

Biosensors are pivotal in monitoring therapeutic drugs, providing swift, sensitive, and precise detection of drug levels in biological samples. This helps in fine-tuning drug doses, ensuring treatment effectiveness, and reducing potential side effects [154,155]. Table 4 reviews some applications of biosensors in therapeutic drug monitoring. By continuously monitoring drug levels in real-time or near-real-time, biosensors enable personalised dosing regimens, enhancing patient care in various clinical settings, including critical care, oncology, psychiatry, and infectious diseases [156]. Additionally, biosensors offer potential cost savings by reducing the need for labour-intensive laboratory techniques and minimising healthcare resource utilisation, thus improving patient outcomes and overall healthcare efficiency [157].

Therapeutic drug monitoring (TDM) involves tracking the plasma concentration of drugs with a narrow therapeutic range. This is achieved through various analytical methods such as HPLC, gas chromatography, and, more recently, biosensors [158,159]. The main problem with drugs with a narrow therapeutic index is that they usually cause toxic effects at the therapeutic dose (an overlap exists between the effectiveness and the toxicity of the drug). For this reason, they require special care during their administration by continuously monitoring and analysing their pharmacokinetic and pharmacodynamic profile [160,161].

**Figure 5:**
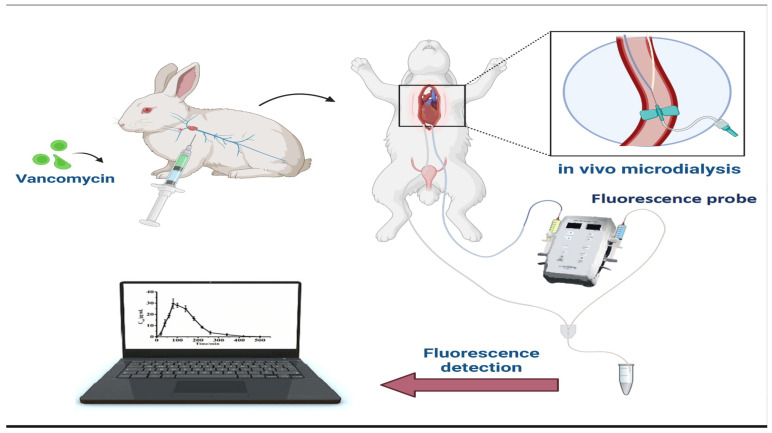
Fluorescence-based biosensor for rapid and sensitive quantitation of vancomycin levels in rabbits.

Vancomycin, a glycopeptide antibiotic, is a robust antibiotic that faces resistance from an increasing number of bacterial strains, often due to inappropriate administration. Furthermore, vancomycin is associated with a variety of side effects, mainly on the ear and the kidneys, therefore requiring close therapeutic drug monitoring [162,163,164]. Mu et al. [165] designed a fluorescence-based biosensor for rapid, highly specific, and highly sensitive quantitation of vancomycin levels in rabbits (Figure 5). The biosensor utilised a vancomycin-specific fluorescent probe, and a tripeptide (L-Lys-D-Ala-D-Ala) linked it to a dansyl chloride group. This analytical technique was coupled with a microdialysis sampling of the biological fluid to avoid blood sampling. This novel biosensor can effectively replace other analytical methods (HPLC and immunoassay) used to measure and monitor vancomycin in humans since it requires no special sample preparation or complicated instrumentation. It holds the potential to be implemented in analysing the pharmacokinetic profile of vancomycin in clinical settings, thus avoiding unnecessary nephrotoxicity, ototoxicity and patient suitability to vancomycin-resistant strains.

Warfarin, frequently prescribed as an anticoagulant with a narrow therapeutic range, requires careful monitoring to prevent excessive bleeding. It acts as a vitamin K antagonist by inhibiting vitamin K epoxide reductase, thus reducing the levels of active vitamin K and impairing the synthesis of vitamin K-dependent clotting factors (II, VII, IX, and X) [166].

Saeedi et al. fabricated a novel electrochemical biosensor based on ion-sensing capability, creating a biosensing electrode that uses tetradodecylammonium chloride as an ion exchanger [167]. In this study, they designed a portable point-of-care testing device that is exceptionally precise and specific for monitoring warfarin levels in patient blood during treatment. The device achieved a detection limit of 1.4 × 10^−5^ M in blood samples. These results highlight the potential of the electrochemical-based biosensor in therapeutic drug monitoring protocols to optimise clinical and therapeutic outcomes effectively [167]. Other laborious methods, such as capillary electrophoresis and HPLC, can test warfarin, but they are costly and time-consuming, making routine therapeutic monitoring of warfarin a challenging task [168,169].

In another study, Ozbek et al. [170] constructed a potentiometric biosensor to monitor the level of valproic acid in human blood. The biosensor showed a fast response time (less than 10 s), good stability, and a detection limit of 9.75 × 10^−7^ mol L^−1^. The results obtained from the biosensor were also compared with the analytical instrument utilised in the hospital where the research was conducted, and the results were compatible. These results suggest that valproate-sensitive biosensors can replace the conventional, time-consuming, and expensive instruments used in hospitals to monitor patients administering drugs with narrow therapeutic indexes such as valproic acid.

Since chemotherapeutic agents also require close monitoring, a study by Alvau et al. designed an irinotecan-sensitive electrochemical biosensor [171]. Irinotecan’s mode of action in cancer therapy is by inhibiting topoisomerase I enzyme. However, irinotecan can also inhibit acetylcholine esterase, which was exploited to design the irinotecan-sensitive biosensor. Their biosensor was based on using cholinesterase and choline oxidase enzymes. The presence of irinotecan hampers the reaction catalysed by those enzymes, and this decrease in the activity can be correlated with the level of irinotecan in the sample [172]. These findings require further research and analysis to provide an irinotecan monitoring test that can be used to monitor patients administering irinotecan.

Another chemotherapeutic drug that requires better analytical instrumentation in its monitoring is doxorubicin. Doxorubicin, like any other chemotherapeutic agent, possesses off-target toxicity. Therefore, optimising the dose administered to cancer patients and monitoring it is vital in doxorubicin therapy [173]. A doxorubicin-sensitive sensor was designed by using a glassy carbon electrode carrying single-wall carbon nanotubes. The single-wall carbon nanotubes were then loaded with double-stranded DNA. The analysis is based on doxorubicin intercalating into the double-stranded DNA grooves and causing an electrochemical change sensed by the electrode. The biosensor showed promising results regarding the sensitivity and selectivity of doxorubicin. The biosensor had a limit of detection of 0.6 nM [174]. A sensitive and selective biosensor for monitoring doxorubicin is promising to be implemented after further validation studies.

Pharmacokinetic analysis is vital in the healthcare system since it allows healthcare experts to monitor how a candidate drug is absorbed, metabolised and finally excreted from the body. Pharmacokinetics focuses on the level of the drug in the body and eventually linking it to the therapeutic outcome [175]. Nowadays, pharmacokinetic data are generated by measuring the concentration of a candidate drug in blood samples. However, these blood-based data can sometimes reflect poor correlation with the actual drug response and hence alter drug monitoring. This is typically seen in cancer therapy [176].

Seo et al. [177] developed an implantable microelectrode array biosensor to generate real-time tumour tissue-based pharmacokinetic data for a more reliable drug efficacy assessment. There was a significant difference between the tumour tissue-based and the blood-based pharmacokinetics. The tumour tissue can be exposed differently than what is reflected or recognised by analysing the blood samples. This study showed that in the future, this implantable biosensor could replace the traditional needle biopsies that are carried out to study drug levels in the tissue.

Another recent study involved designing a multifunctional biosensor to assess the efficacy of a novel epidermal growth factor receptor tyrosine kinase inhibitor (AC0010). The sensor was utilised to generate electrical impedance data that were processed to reflect tumour cell growth and death and assess the drug’s efficacy. To ensure that the biosensor generates reliable data, results were compared to those obtained from CKK8 assays. In addition, the multifunctional biosensor was also used successfully to monitor the electrophysiological and beating activity of the myocardial cells [178].

**Table 4:** Biosensors applications in therapeutic drug monitoring (TDM).

Drug Monitored	Biosensor	Transducer	Matrix	LOD	Linearity Range	Ref.
Amoxicillin	Enzyme Biosensor with Microfiber Interferometer (MFI) and Fiber Gratings (FBGs) Power Variation	Optical	Deionised water, real food, urine samples	0.04 nM	0.01–100 nM	[179]
Tenofovir (TFV)	Alkaline phosphatase (ALP) Enzyme with BaTiO_3_ Nanoparticles	Electrochemical	Human blood serum	0.09 nM	N/A	[180]
Kanamycin	Ru@MOF/Ag^+^-Dependent DNAzyme ECL Biosensor	Electrochemiluminescence	Seawater, Milk	13.7 pM	30 pM–300 μM	[181]
	DNA Aptamer-Modified Portable Gold Electrode Biosensor	Electrochemical	Environmental water samples	0.40 μmol/L	1–1000 μmol/L	[182]
	Thionine functionalised graphene and hierarchical nanoporous (HNP) PtCu Aptasensor	Electrochemical	Animal-derived food	0.42 pg mL^−1^	5 × 10^−7^– 5 × 10^−2^ μg mL^−1^	[183]
Streptomycin	Colorimetric Casein hydrolysate peptides-functionalised silver nanoparticles (CHPs@AgNPs)	Colourimetric	Tap water, Dairy whey	~98 nM (tap water) ~56 nM (dairy whey)	200–650 nM (tap water); 100–700 nM (dairy whey)	[184]
FK506	Spiky Fe_3_O_4_@SiO_2_@Ag flower magnetic superstructure and hollow Ag@Au superstructure enhanced SERS biosensor	Magnetic/Plasmonic	Blood of transplant patients	0.33 ng/mL	0.5–20 ng/mL	[185]
Vincristine	DNA (ds-DNA), polypyrrole (PP), peony-like CuO:Tb^3+^ nanostructure (P-L CuO:Tb^3+^ NS)	Electrochemical	Pharmaceutical preparations, biological fluids	0.21 nM	1.0 nM–400.0 μM	[186]
Imatinib (IMA)	DNA/AuPt/p-L-Met Coated SPE Electrochemical DNA Biosensor	Electrochemical	Human serum, pharmaceutical samples	0.18 nM	2.33–80 nM	[187]
Erlotinib (ERL)		Electrochemical	Human serum, pharmaceutical samples	0.009 nM	0.032–1.0 nM	
Doxorubicin (DOX)	Gold nanoparticles (AuNPs) and DNA tetrahedron (TDN) nanoprobe bifunctional glassy carbon electrode	Electrochemical	Human serum, cell lysate	0.3 nM	1.0 nM–50 μM	[188]
Daunorubicin (DNR)	Upconversion nanoparticles (UCNPs) as energy donor and herring sperm DNA (hsDNA) biosensor.	Luminescence	Biological fluids	0.60 μg·mL^−1^	1–100 μg·mL^−1^	[189]

## 6. Concentration Ranges and S/N Ratios for Key Biomarkers

In addition to detection limits, it is crucial to consider the concentration ranges and signal-to-noise (S/N) ratios in healthy subjects for accurate detection of target molecules. These parameters are vital because they provide a benchmark against which abnormal levels can be identified, enhancing the reliability and sensitivity of biosensors in clinical diagnostics and monitoring. Understanding the typical concentration ranges of target molecules in healthy individuals helps in setting accurate thresholds for detecting deviations that may indicate disease or other health conditions. Similarly, the signal-to-noise (S/N) ratio is essential to distinguish true signals from background noise, ensuring precise measurements [190,191]. Table 5 provides detailed information on the concentration ranges and S/N ratios for various target molecules in healthy subjects, illustrating their clinical relevance and importance for biosensor performance.

**Table 5:** Concentration ranges and signal-to-noise ratios for some target molecules in healthy subjects. S/N are only for demonstration purposes since it is highly system specific.

Target Molecule	Healthy Subject Concentration Range	S/N Ratio	Clinical Relevance	Ref.
Cardiac Troponin I (cTnI)	1 pg/mL–100 ng/mL	10:1	Indicative of myocardial infarction	[192]
Anti-dsDNA Autoantibodies	1–200 IU/mL	10:1	Marker for rheumatoid arthritis	[193]
Glucose	3.9–5.5 mmol/L	15:1	Monitoring diabetes mellitus	[194]
Prostate-Specific Antigen (PSA)	0–4 ng/mL	15:1	Screening for prostate cancer	[195]
Cholesterol (Total)	<200 mg/dL	10:1	Assessing cardiovascular health	[196]
C-Reactive Protein (CRP)	0.8–3.0 mg/L	10:1	Indicator of inflammation	[197]
Hemoglobin A1c (HbA1c)	4–5.6%	15:1	Long-term glucose level monitoring	[198]
Thyroid-Stimulating Hormone (TSH)	0.4–4.0 mIU/L	10:1	Assessing thyroid function	[199]

These S/N ratios are approximate and are based on typical biosensor performance. They serve to illustrate the importance of distinguishing true signals from background noise, which is critical for the accuracy and reliability of biosensor measurements in clinical diagnostics.

## 7. Research Challenges for Future Applications

Biosensor technology has undoubtedly undergone remarkable advancements in healthcare. Yet, it is imperative to acknowledge that several formidable research challenges persist on the journey toward its widespread adoption and further advancement. Addressing these multifaceted challenges is essential and pivotal in unlocking the full potential of biosensors to revolutionise patient outcomes and reshape the landscape of healthcare delivery. By confronting these challenges head-on, the healthcare industry can harness the transformative power of biosensors to enhance diagnostic accuracy, improve disease management strategies, and ultimately elevate the standard of patient care to unprecedented heights.

### 7.1. Sensitivity, Specificity, and Reproducibility

The effectiveness of biosensors in healthcare heavily relies on their sensitivity, specificity, and reproducibility. Achieving high sensitivity ensures the detection of minute biomarker concentrations, which is crucial for early disease detection and monitoring. Specificity is equally vital to accurately identifying the target analytes amidst complex biological samples. Reproducibility, on the other hand, ensures consistent performance across various conditions and settings, minimising variability in results. However, challenges persist in maintaining these parameters due to factors such as sample matrix variations, environmental influences, and user-related variability. Future research endeavours must prioritise refining detection mechanisms, optimising sensor materials, and standardising protocols to enhance the reliability and robustness of biosensor performance [200].

### 7.2. Integration with Electronic Health Records (EHR) and Data Security

Seamless integration of biosensor-generated data with Electronic Health Records (EHR) holds immense promise for advancing personalised medicine and data-driven healthcare decision-making. However, achieving this integration is fraught with challenges. Interoperability issues between different systems and data formats hinder smooth data exchange, while concerns regarding data privacy and cybersecurity pose significant risks to patient confidentiality. Establishing robust data exchange standards, implementing encryption protocols, and ensuring compliance with stringent privacy regulations are imperative steps towards safeguarding sensitive patient information. By addressing these concerns, healthcare providers can harness the full potential of biosensor data while upholding patient privacy and security [201,202].

### 7.3. Potential Breakthroughs and Future Directions

Despite the existing research challenges, the future of biosensor technology in healthcare is marked by promising avenues for innovation. Advances in nanotechnology, materials science, and bioinformatics offer unprecedented opportunities for revolutionising biosensor design and functionality [203]. Nanomaterials with unique properties can enhance sensitivity and specificity, while multiplexed biosensor arrays enable simultaneous detection of multiple analytes, paving the way for comprehensive disease profiling [204]. Integrating artificial intelligence (AI) and machine learning algorithms holds immense potential for enhancing data analysis, pattern recognition, and predictive modelling, facilitating early disease detection and personalised treatment strategies. Moreover, the emergence of point-of-care molecular diagnostics, coupled with microfluidics and lab-on-a-chip technologies, promises to democratise healthcare delivery by enabling rapid, cost-effective, and accessible diagnostics, particularly in resource-limited settings [205].

## 8. Conclusions

In conclusion, biosensor technologies are revolutionising healthcare diagnostics by enabling precise and rapid detection of disease biomarkers and facilitating effective therapeutic drug monitoring. This review has explored the diverse types of biosensors, their mechanisms, and their specific applications in healthcare. The integration of nanotechnology, wearable devices, and miniaturisation trends has demonstrated significant potential to transform patient care. Despite sensitivity, specificity, reproducibility, and data security challenges, strategic solutions and ongoing innovations are paving the way for enhanced global healthcare outcomes. By fostering interdisciplinary collaboration and driving technological advancement, biosensors are poised to play an increasingly pivotal role in modern medicine.
